# Medicinal Activities and Nanomedicine Delivery Strategies for *Brucea javanica* Oil and Its Molecular Components

**DOI:** 10.3390/molecules25225414

**Published:** 2020-11-19

**Authors:** Bo Kyeong Yoon, Zheng Yi Lim, Won-Yong Jeon, Nam-Joon Cho, Jeong Hoon Kim, Joshua A. Jackman

**Affiliations:** 1 School of Chemical Engineering and Biomedical Institute for Convergence at SKKU (BICS), Sungkyunkwan University, Suwon 16419, Korea; bky0622@skku.edu (B.K.Y.); limz0136@e.ntu.edu.sg (Z.Y.L.); powerwy@skku.edu (W.-Y.J.); 2 School of Materials Science and Engineering, Nanyang Technological University, Singapore 637553, Singapore; njcho@ntu.edu.sg; 3 Omni Colab Corporation, Suwon 16229, Korea; 4 Department of Health Sciences and Technology, Samsung Advanced Institute for Health Sciences and Technology, Sungkyunkwan University, Seoul 06351, Korea; jeongkim@skku.edu

**Keywords:** *Brucea javanica* oil, quassinoid, medicinal activity, nanomedicine, emulsion, liposome, spongosome, drug delivery

## Abstract

*Brucea javanica* oil (BJO) is widely used in traditional Chinese medicine to treat various types of cancer and inflammatory diseases. There is significant interest in understanding the medicinal activities of BJO and its molecular components, especially quassinoids, and in exploring how they can be incorporated into nanomedicine delivery strategies for improved application prospects. Herein, we cover the latest progress in developing different classes of drug delivery vehicles, including nanoemulsions, liposomes, nanostructured lipid carriers, and spongosomes, to encapsulate BJO and purified quassinoids. An introduction to the composition and medicinal activities of BJO and its molecular components, including quassinoids and fatty acids, is first provided. Application examples involving each type of drug delivery vehicle are then critically presented. Future opportunities for nanomedicine delivery strategies in the field are also discussed and considered within the context of translational medicine needs and drug development processes.

## 1. Introduction

The field of traditional Chinese medicine (TCM) often incorporates natural products from plant, animal, and mineral sources as medicinally active ingredients for applications such as anticancer, anti-inflammatory, and antimicrobial therapy [1]. Given the use of natural products and historical precedent, there is broad interest in exploring the potential advantages of TCM as an alternative to modern medicine, and potential benefits include greater efficacy, lower costs, fewer side effects, and perceived safety when used judiciously [2].

**Figure 1:**
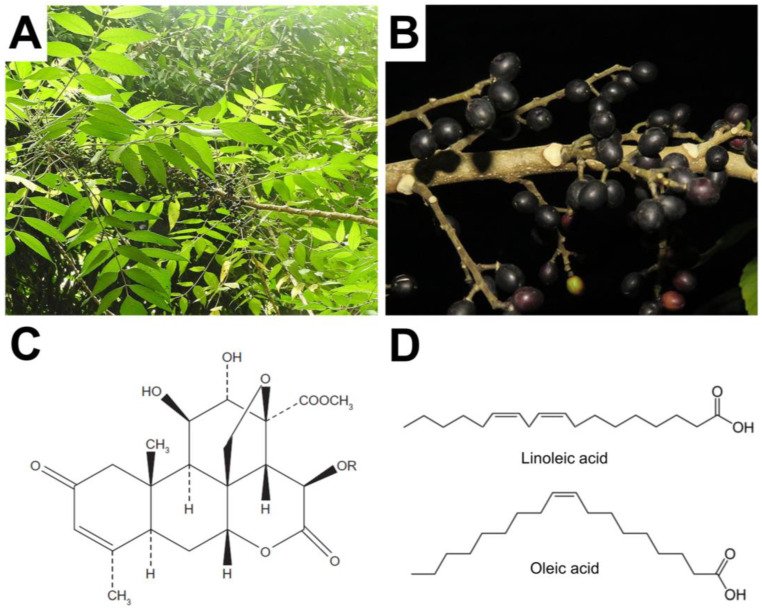
(**A**) Picture of *Brucea javanica* plant (Credit: Yercaud Elango under CC BY-SA 4.0 license); (**B**) Picture of *Brucea javanica* fruit (Credit: Ruben C. J. Lim under CC BY-NC-SA 2.0 license); (**C**) Chemical structure of a quassinoid molecule, where R denotes a generic substituent group; (**D**) Chemical structures of linoleic acid and oleic acid molecules. Reproduced with permission from Ref. [4].

As shown in Figure 1A, *Brucea javanica* (L.) Merr is a species of evergreen plant shrub from the Simaroubaceae family that grows abundantly throughout Southern China and Southeast Asia [3]. The bitter fruit of *B. javanica* is oval-shaped, solid, and has a typical length and diameter of around 8 mm and 5 mm, respectively [4] (Figure 1B). Listed in the official Chinese Pharmacopoeia that describes TCM ingredients, the *B. javanica* fruit is often used to treat medical illnesses such as intestinal inflammation, diarrhea, malaria, and different types of cancer [5,6]. It has been claimed that the fruit is also useful to treat ailments such as abdominal pain, hemorrhoids, hyperkeratosis, and ulcers [7].

The fruit and especially its seed oil—termed *B. javanica* oil (BJO)—contain medicinally active components, including quassinoids as well as fatty acids such as oleic acid and linoleic acid [4,8]. The chemical structures of these compounds are presented in Figure 1C,D. Quassinoids have a tetracyclic triterpene structure that consists of three six-membered carbon rings and a six-membered lactonic ring, which has a variable substituent group that defines the quassinoid identity [9]. Notably, quassinoids are understood to be found exclusively in plants from the Simaroubaceae family [10], a feature that has heightened interest in BJO due to the wide range and high potency of medicinal activities that quassinoids can exhibit, as discussed below.

While BJO and its molecular components are medicinally active, they have a poor solubility in aqueous solutions, which has motivated the development of *B. javanica* oil emulsion (BJOE) systems that enable improved solubility [11]. BJOE samples are composed of oil extracts isolated from *B. javanica* fruit together with a naturally sourced lipid emulsifier [12]. To date, intravenously administered BJOE has been used clinically, including in China, as a standalone treatment [13] and in combination with radiotherapy [14] or chemotherapeutic drugs [15] to improve cancer treatment efficacy and enhance immune functions [16]. However, BJOE still has shortcomings, such as a relatively low bioavailability of medicinally active compounds, physicochemical stability issues, and potential administration side effects such as blood vessel irritation and adverse immune reactions [17,18].

Such challenges have spurred ongoing efforts to develop innovative nanomedicine strategies that harness the medicinal activity of BJO and its molecular components with nanoparticle carriers. Key examples of promising nanostructures in various stages of development include nanoemulsions, liposomes, nanostructured lipid carriers, and spongosomes, which can help to improve bioavailability, controlled release, physicochemical stability, drug loading, and encapsulation efficiency. The objective of this review is to cover the latest progress in the field, starting with an overview of the pharmacological properties of BJO and its molecular components, followed by a critical presentation of various types of cutting-edge nanomedicine strategies and how they are being utilized to deliver BJO and purified quassinoids.

## 2. Overview of *Brucea javanica* Oil and Molecular Components

**Figure 2:**
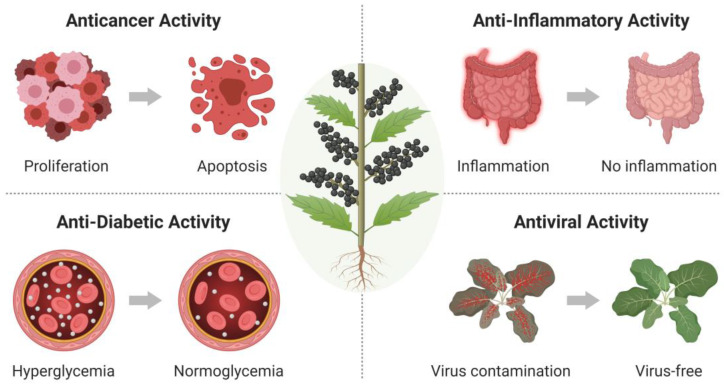
Schematic illustrations of four representative medicinal activities of BJO.

BJO is mainly extracted from *B. javanica* seeds, and the major molecular components are various fatty acids and fatty acid derivatives, including around 63% oleic acid and 21% linoleic acid in free and mono-, di- and triglyceride forms, along with medicinally active quassinoids in lower concentrations [19]. While oleic acid and linoleic acid constitute a large fraction of BJO contents by mass, quassinoids are present in lower concentrations but exhibit a rich diversity and can have highly potent biological activities. To date, over 100 quassinoids have been identified in BJO [20]. Certain classes of quassinoids, such as bruceines, brusatols, and bruceosides, typically exhibit high levels of biological activity [21]. In this section, we introduce four representative types of medicinal activity that BJO and its molecular components exhibit. Schematic illustrations of these activities are presented in Figure 2.

### 2.1. Anticancer Activity

Since BJO has been shown to have clinical prospects as a cancer treatment option, there have been intensive experimental efforts to investigate the anticancer activity of BJO. Accordingly, BJO has been shown to inhibit leukemia [22], liver [23], and lung [24] cancer cells in vitro. BJO can exhibit anticancer activity by multiple mechanisms, including by inducing cancer cell apoptosis [25] and inhibiting the function of enzymes (e.g., DNA topoisomerases) related to chemotherapeutic drug resistance mechanisms [26]. It has been discussed how BJO-induced cancer cell apoptosis can involve the upregulated expression of caspase enzymes associated with programmed cell death and the downregulated expression of other important biological machinery components [27].

Various molecular components of BJO are believed to contribute to anticancer activity. For example, linoleic acid is reported to inhibit the cyclooxygenase-2 (COX-2) enzyme that is related to tumor cell invasiveness and angiogenesis [28,29]. In addition, oleic acid can induce cancer cell apoptosis and autophagy [30]. Importantly, many quassinoids from BJO are also well known for exhibiting therapeutic activity to treat bone [31], breast [32], pancreatic [33], lung [34], and liver [35] cancers in vitro. For example, Wang et al. found that the quassinoid bruceine D (BD) induced apoptosis of osteosarcoma cells along with inhibiting cancer cell proliferation and migration [31]. These inhibitory activities involved BD blocking a cell signaling pathway that was implicated in cancer cell malignancy [36]. In addition, BD has been found to inhibit hepatocellular carcinoma by inhibiting signaling pathways related to liver carcinogenesis and disease progression [35]. Quassinoids are typically more potent than fatty acids, with 50% inhibitory concentration (IC_50_) values down to the nanomolar level [37], and hence most therapeutic focus has been on quassinoids [38,39].

### 2.2. Anti-Inflammatory Activity

Inflammatory bowel diseases such as Crohn’s disease and ulcerative colitis can affect the small and large intestines and cause a wide range of symptoms such as bloody diarrhea, vomiting, and abdominal pain [40]. Hence, there is interest in developing therapeutic strategies to modulate the activity of inflammatory cells and signaling pathways. Such objectives also demand the delivery of therapeutics to disease sites such as the colon and rectum in the case of ulcerative colitis, whereas Crohn’s disease mainly causes inflammation of the gastrointestinal tract, especially in the small intestine [41]. Mechanistically, the nuclear factor-κB (NF-κB) family of transcription factors is an important mediator of innate and immune responses, which controls the expression of proinflammatory cytokines and chemokines and modulates immune cell activation [42]. Aberrant NF-κB signaling is also a factor in cancer initiation [43]. In patients with ulcerative colitis, the NF-κB pathway has a heightened activity, which can lead to an upregulated cytokine expression that contributes to increased levels of inflammation [44].

Huang et al. reported that BJOE could have therapeutic benefits in an experimental rat model of Crohn’s disease and inhibited the activation of the NF-κB pathway, resulting in decreased levels of proinflammatory cytokines and increased levels of anti-inflammatory cytokines [41]. In a mouse model of ulcerative colitis, it was also reported that BJOE exhibited therapeutic activity by inhibiting the NF-κB pathway [45]. Li et al. further identified that BJOE administration also helped to reduce the incidence of inflammation-related gastric ulcers in mouse and rat models in a dose-dependent manner [46]. These experimental results have been obtained using BJOE in various animal models, while there have also been efforts to better understand which molecular components are responsible for therapeutic effects. For example, it has been identified that components such as the quassinoid brusatol (BR) can be useful for treating inflammatory diseases [47]. Indeed, a wide range of quassinoids, including BR, have been observed to inhibit induced inflammation and arthritis in rat models [48]. The oral administration of oleic or linoleic acids in rats has also been observed to decrease the production of inflammatory mediators by primary macrophages [49], suggesting that anticancer properties might be related to preventing excessive inflammation.

### 2.3. Anti-Diabetic Activity

While it has long been suggested that BJO is useful for treating diabetes mellitus [50], more recent work has investigated which molecular components in BJO are responsible for antidiabetic activity. Ablat et al. tested fractions of a BJO extract—which contained different molecular components that had been separated by using various solvents with distinct polarities—to evaluate the therapeutic activity in a rat model of diabetes and identified that certain fractions could reduce blood glucose levels along with more favorable insulin and glycogen results and decreased levels of oxidative stress markers and inflammation [51]. Oxidative stress is associated with a diabetic phenotype, including a reduced glucose tolerance and insulin resistance, so reducing its prevalence is beneficial from a treatment perspective [52].

NoorShahida et al. extracted various fractions of medicinally active compounds from BJO and observed that the oral or intraperitoneal administration of BJO fractions could reduce blood glucose levels in normoglycemic mice [53]. Further investigation revealed that the extracted fraction that contained the BD quassinoid had the greatest effects, which prompted the isolation and further examination of BD. Dose-dependent reductions in the blood glucose levels of normoglycemic mice upon intraperitoneal administration of BD were observed. Notably, to achieve a reasonable solubility, the BJO fractions and isolated quassinoids had to be formulated with the organic solvent dimethylsulfoxide (DMSO) or with poly(ethylene glycol) (PEG) polymer chains, highlighting the need to further develop effective delivery strategies, as discussed below.

### 2.4. Antiviral Activity

Plant viruses are a major threat to agricultural production [54] and have led to the exploration of quassinoids from BJO as a potential antiviral solution. Crude extracts of BJO were first reported to inhibit tobacco mosaic virus (*TMV*) infection and replication [55]. A follow-up study tested different BJO fractions and identified that the BD quassinoid had a particularly high antiviral activity against *TMV*, could be used as a foliar spray to inhibit the systemic *TMV* infection of plants, and also inhibited potato virus Y (*PVY*) and cucumber mosaic virus (*CMV*) [56]. It has been suggested that quassinoids might help to enhance plant resistance to viral infection, which could also potentially relate to immunomodulatory activities [57].

Using a leaf-based assay, Ryu et al. further investigated the antiviral properties of various quassinoids to inhibit pepper mottle virus (*PepMOV*), which affects pepper plants (*Capsicum* spp.) and can impair pepper production quality and yield [58]. It was discovered that a BJO extract and various quassinoids therein, including brucein A, inhibited the *PepMOV* infection of plant leaves based on a fluorescence reporter assay, in which case *PepMOV*-infected leaves exhibited fluorescence under ultraviolet (UV) light. In marked contrast to this result, leaves treated with BJO extract or brucein A quassinoid did not exhibit fluorescence, indicating an effective antiviral activity. Real-time polymerase chain reaction (RT-PCR) and Western blot data further indicated the lack of viral coat protein for the treated samples, in alignment with the negative control data without virus and in contrast to the virus-only positive control data.

In addition to plant viruses, certain quassinoids have also been reported to inhibit certain human viruses, such as human immunodeficiency virus (*HIV*) along with herpes simplex, Semliki forest, and Coxsackie viruses [59,60].

## 3. Nanomedicine Delivery Strategies

BJOE formulations have demonstrated clinical utility as a means of utilizing the medicinal activities of BJO for anticancer applications. However, BJOE formulations have drawbacks, such as low bioavailability, physicochemical stability issues, and potential administration side effects, as described above. To overcome these issues, nanoparticle carriers have been widely explored to improve solubility and formulation properties, increase bioavailability, and extend circulation time [4]. Extensive progress has been achieved in recent years with various classes of self-assembled nanostructures, which have been utilized to encapsulate not only BJO but also purified molecular components with a high medicinal activity, such as particular quassinoids. The latest results are described below and organized according to nanostructure class. Where applicable, the results are divided between nanomedicine strategies that utilize whole BJO vs. purified quassinoids extracted from BJO.

### 3.1. Nanoemulsions

There have been extensive efforts aimed at developing oil-in-water emulsions that are composed of a mixture of BJO or quassinoids plus different types and amounts of surfactants and cosurfactants. Depending on the specific preparation conditions, the nanoemulsions have typical particle sizes of around 10 to 300 nm [61,62].

#### 3.1.1. BJO Encapsulation

Yang et al. reported the development of a BJO nanoemulsion formulation with an improved safety profile for intravenous administration [63]. A phase diagram of BJO and isopropyl myristate (IPM) plus different surfactants and cosurfactants in varying ratios was constructed to distinguish systems that formed nanoemulsions vs. those that formed microemulsions. Transmission electron microscopy (TEM) images of the lead composition showed spherical particles with a diameter of around 24 nm, which is indicative of nanoemulsions. In vitro hemolysis testing further confirmed that the nanoemulsions did not cause hemolysis, while some hemolysis was observed at high concentrations of BJOE tested in parallel. Vein injection testing in rabbits further showed that the nanoemulsions had more favorable outcomes than BJOE, with a lower likelihood of vein irritation. The nanoemulsions also exhibited a more potent in vitro cytotoxicity against a human cervical cancer cell line than BJOE.

Wang et al. reported another BJO nanoemulsion formulation that consisted of oil-in-water emulsions with a diameter of approximately 41 nm [64]. A 70-mg/kg dose of nanoemulsions or a refined BJO control sample was orally administered to beagle dogs, and pharmacokinetic studies revealed that the nanoemulsions had a nearly twofold greater maximum BJO concentration and circulation half-life. These findings supported that the nanoemulsion formulation enabled greater oral bioavailability. In vitro cancer cell cytotoxicity testing showed that the nanoemulsions also had an appreciably more potent inhibitory activity against multiple cancer cell lines than a commercial BJOE sample. The improved activity was suggested to be the result of the nanoemulsion’s size, which might have increased the cancer cell contact area. In a cancer mouse model bearing sarcoma tumors, the intragastric administration of the nanoemulsions and BJOE showed that the nanoemulsions had a superior treatment performance, as indicated by a greater reduction in the tumor weight.

It is also possible to develop BJO nanoemulsions with liquid or solid granule formulations of around ~35 nm in diameter, which can be useful for oral delivery applications [65]. Huang et al. also reported the successful encapsulation of BJO in a solidified form, and the BJO nanoparticles had an average diameter of around 277 nm [66]. Importantly, the nanoparticles exhibited a significantly greater inhibitory activity against lung and prostate cancer cell lines in vitro than BJOE, and they also inhibited cancer cell invasion. There has also been exploration of cationic emulsions incorporating BJO and chitosan that had an average particle diameter of around 42 nm and that exhibited a more effective treatment than BJOE in a mouse model of lung cancer, in addition to exhibiting a synergistic activity together with chemotherapeutic drugs [67].

#### 3.1.2. Quassinoid Encapsulation

In addition to using compositionally diverse BJO, there have recently been more focused efforts to develop nanoemulsions containing highly active quassinoids extracted from BJO. Nanoemulsions containing the BR quassinoid were used to treat ulcerative colitis in mice [68]. The nanoemulsions had a diameter of around 26 nm and had a 95% BR encapsulation efficiency, and they were tested in parallel with an aqueous BR formulation control. The aqueous formulation was prepared using a sodium carboxymethyl cellulose solution, although there was a poor solubility of BR and high turbidity. The bioavailability levels of BR in the nanoemulsions and aqueous suspension were measured in rats, and the nanoemulsion delivery platform enabled a longer circulation half-life and higher maximum concentration of BR in plasma. Treatment with 0.25-, 0.5-, and 1.0-mg/kg doses of orally administered BR in nanoemulsions showed a dose-dependent efficacy in terms of clinically relevant disease parameters, tissue morphology, and inflammatory markers in a mouse model of chemically induced colitis.

**Figure 3:**
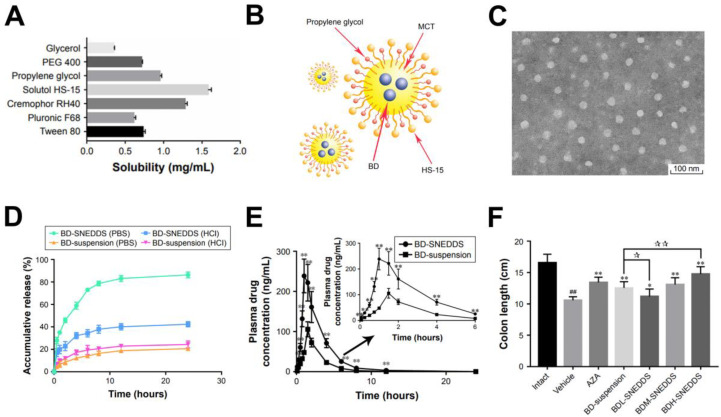
(**A**) Solubility of bruceine D (BD) in different excipients; (**B**) Schematic diagram of BD in the self-nanoemulsifying drug delivery system, which was termed BD-SNEDDS and consisted of BD in medium-chain triglyceride (MCT) oil and encapsulated within PEG-15-hydroxystearate (HS-15) and propylene glycol cosurfactants; (**C**) Transmission electron microscopy (TEM) image of BD-SNEDDS nanoemulsions; (**D**) In vitro release profiles of BD in BD-SNEDDS nanoemulsions and in an aqueous suspension in simulated intestinal fluid comprising phosphate-buffered saline (PBS) (pH 6.8) and in simulated gastric fluid comprising HCl (pH 1.2) conditions; (**E**) Mean concentration of BD in rat plasma at different time points following oral administration of BD-SNEDDS nanoemulsions and aqueous BD. The inset shows a magnified view between 0 and 6 h postadministration. ** indicates *p* < 0.01 vs. BD-suspension group; (**F**) Colon lengths of rats in an experimental colitis model with the following test groups: Intact: no treatment; Vehicle: treatment without drug; AZA: treatment with azathioprine; BD-suspension: BD in PBS; BDL-SNEDDS, BDM-SNEDDS, and BDH-SNEDDS are treatments with low, medium, and high doses of BD-SNEDDS, respectively. ^##^ indicates *p* < 0.01 vs. Intact group. * and ** indicate *p* < 0.05 and *p* < 0.01 vs. Vehicle group, respectively. ^☆^ and ^☆☆^ indicate *p* < 0.05 and *p* < 0.01 vs. BD-suspension group, respectively. Reproduced with permission from [69].

Dou et al. reported another nanoemulsion delivery platform for quassinoids, which they termed a self-nanoemulsifying drug delivery system [69]. The nanoemulsions were used to encapsulate BD quassinoid and were orally administered to treat ulcerative colitis in rats. Different excipients were tested as candidates to improve the BD solubility in water, and Solutol HS-15 and propylene glycol were identified as high-performing ones (Figure 3A). Various oils were also tested to improve the BD solubility, and a medium-chain triglyceride (MCT) oil was the best performing one. As such, nanoemulsions comprising BD in a mixture of MCT oil, Solutol HS-15, and propylene glycol were fabricated (Figure 3B,C). The BD nanoemulsions had typical diameters of around 20 nm and remained stable after various processing conditions such as heating, cooling, and repeated freeze-thawing, as well as upon dilution in aqueous solutions with different pH conditions. In vitro release profile testing in simulated intestinal (pH 6.8) and gastric (pH 1.2) fluid conditions showed that the BD nanoemulsions enabled a greater release in near-neutral pH conditions, with a total release of around 86% over an approximately 12-h time period (Figure 3D).

The in vivo pharmacokinetics of BD in the nanoemulsions vs. free BD in an aqueous sodium carboxymethyl cellulose solution were compared upon oral administration of a 3-mg/kg dose in rats (Figure 3E). The nanoemulsion formulation yielded a more than twofold higher maximum concentration of BD in plasma compared to the aqueous BD suspension, along with a longer circulation half-life of around 2.6 h vs. 1.5 h. In a rat model of chemically induced colitis, 0.75-, 1.5-, and 3.0-mg/kg doses of the BD nanoemulsions were orally administered and improved clinically relevant disease parameters, for instance through reductions in diarrhea, bloody stool, and colon mucosal damage as well as the prevention of colon length shortening (Figure 3F). The results were comparable to or exceeded those of azathioprine (AZA), which is a medication used to treat colitis and various other ailments. The BD nanoemulsions also reduced the levels of proinflammatory cytokines, increased the levels of anti-inflammatory cytokines, and suppressed oxidative stress associated with colon inflammation.

### 3.2. Liposomes

Liposomes are another useful nanoparticulate delivery system and are comprised of spherical lipid bilayers that can entrap hydrophilic molecules in the aqueous interior and/or hydrophobic molecules in the lipid bilayer [70]. Compared to nanoemulsions, one potential advantage of liposomes is that they have a greater loading capacity, which means that they require the use of fewer surfactant-like molecules and can hence have a greater biocompatibility [71]. Moreover, BJO-loaded liposomes can be functionalized with molecular ligands that bind to receptors that are overexpressed on cancer cells, and one such system based on targeting abundantly expressed receptors present on ovarian cancer cell surfaces led to an increased intracellular uptake of BJO-loaded liposomes, an enhanced cancer cell inhibition, and a greater apoptosis [72].

**Figure 4:**
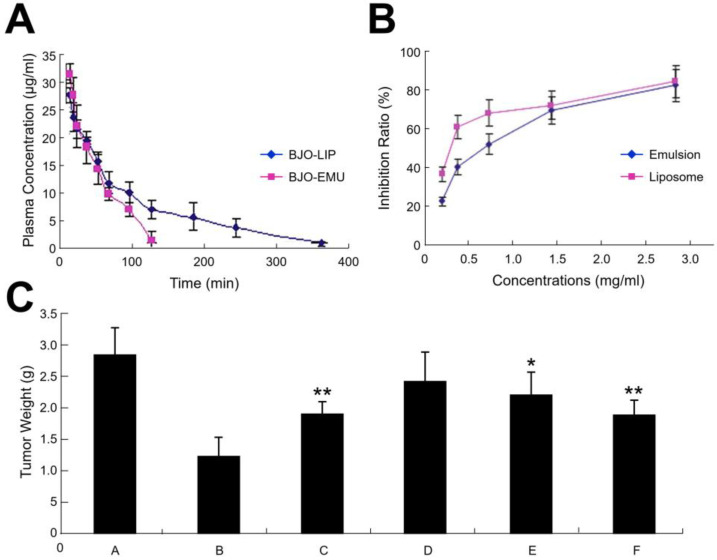
(**A**) Concentration of *Brucea javanica* oil (BJO) in mouse plasma at different time points after the intravenous administration of BJO-containing liposomes (BJO-LIP) or emulsions (BJO-EMU); (**B**) Concentration-dependent inhibitory effects of BJO-containing liposomes and emulsions on human liver tumor cells in vitro; (**C**) Effects of BJO-containing liposome and emulsion treatments on the post-treatment weight of tumors in a mouse model. Treatment conditions and doses are as follows: A: Control, B: Cyclophosphamide at 25 mg/kg, C: Emulsions at 180 mg/kg, D: Liposomes at 60 mg/kg, E: Liposomes at 120 mg/kg, F: Liposomes at 180 mg/kg. * and ** indicate *p* < 0.05 and *p* < 0.01 vs. Control group A, respectively. Adapted with permission from [17].

Cui et al. reported the development of a BJO liposome system comprising 80% phospholipid, 12% cholesteryl, and 8% BJO [17]. The liposomes had an average diameter of around 108 nm. Notably, the BJO liposome sample had a much less acute toxicity than a BJOE sample upon intravenous administration in mice. Pharmacokinetic studies were also conducted for intravenously administrated BJO liposome and BJOE samples, and the liposomes had an appreciably longer circulation time (Figure 4A). In vitro testing of the BJO liposomes and BJOE against a human liver tumor cell line also showed that the liposomes had a more potent inhibitory activity (Figure 4B). A similar performance was also observed in a mouse model with Lewis lung cancer, and the treatment outcomes were judged by tumor weight. An intravenous administration of BJO liposomes led to significant reductions in the tumor weight at 120- and 180-mg/kg doses, while BJOE only caused a reduction at a 180-mg/kg dose (Figure 4C).

Yue et al. also investigated the effects of BJO liposomes on cancer cells in vitro and reported a greater inhibitory activity compared to that of a BJOE sample tested in parallel [73]. The higher potency of the BJO liposomes also translated into better success at preventing intrahepatic metastasis in tumor-bearing mice in vivo. A fivefold lower dose of intraperitoneally administered BJO-loaded liposomes inhibited intrahepatic metastasis by almost 99%, while the BJOE sample only demonstrated a 75% inhibition.

### 3.3. Nanostructured Lipid Carriers

Other lipid nanostructures such as nanostructured lipid carriers (NLCs) have also been explored and have an imperfect crystalline structure that enables high drug loading [74]. Hence, NLCs have been explored for the encapsulation of BJO by itself and in combination with other chemotherapeutic drugs for anticancer applications.

**Figure 5:**
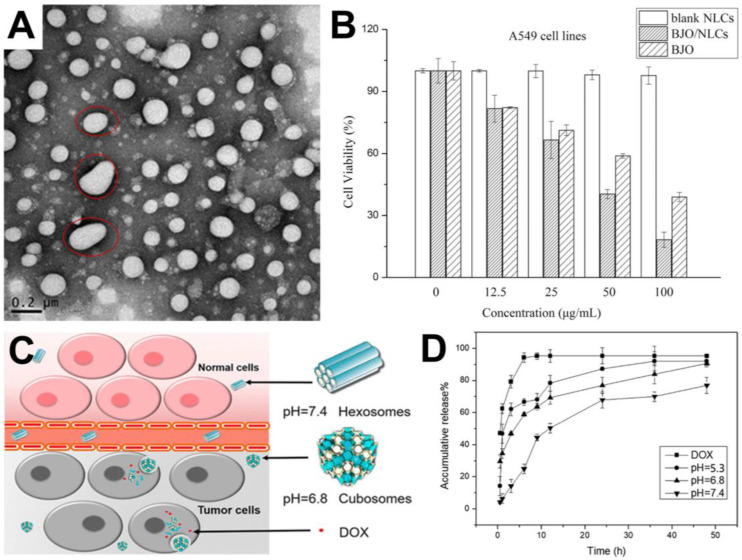
(**A**) Transmission electron microscopy (TEM) image of nanostructured lipid carrier (NLC)-encapsulated *Brucea javanica* oil (BJO) in phosphate-buffered saline (PBS) (pH 7.4). Red circles indicate representative NLC particles with spherical or elongated shapes; (**B**) Effects of blank NLCs as well as free and NLC-encapsulated BJO on the viability of human lung cancer cells after 48 h of incubation. Reproduced with permission from [75]. (**C**) Schematic illustration of pH-dependent changes in the lipid crystalline nanoparticle (LCNP) morphology for anticancer applications. (**D**) pH-dependent in vitro release of doxorubicin (DOX) from LCNPs coloaded with DOX and BJO. Reproduced with permission from [79].

Lv et al. developed an NLC carrier based on a combination of solid- and liquid-phase lipids and other materials to encapsulate BJO [75]. The mixture was processed using high-pressure homogenization, resulting in the formation of BJO-loaded NLCs with a typical diameter of around 182 nm. The NLCs exhibited a high physical stability after a 30-day storage, and transmission electron microscopy (TEM) identified that the NLCs were mainly spherical or moderately elongated (Figure 5A). Small angle X-ray scattering (SAXS) experiments supported the presence of a dense particle core and the loading of BJO within the particles rather than on the particle surface. These morphological observations were consistent with the measured encapsulation efficiency and drug-loading values of 99% and 10%, respectively. These values indicated a high loading performance, which was attributed to defects within the crystal structure [76,77] and a suitable lipid environment [78] for entrapping BJO.

NLC loading markedly slowed the BJO release, and slightly more than 50% of BJO was gradually released over a roughly four-day period, as opposed to a rapid release within 10 h for free BJO. In vitro cell cytotoxicity testing against human lung cancer cells further demonstrated that NLCs without BJO did not have an inhibitory activity, while free and NLC-encapsulated BJO exhibited a dose-dependent inhibition (Figure 5B). The NLC-encapsulated BJO had the greatest inhibitory potency and caused the most extensive changes in the cell morphology, along with inducing higher rates of cell apoptosis.

Expanding on this concept, Li et al. developed pH-responsive lipid nanoparticles with liquid crystalline properties, which were loaded with BJO and doxorubicin (DOX), a widely used anticancer medication [79]. The lipid nanoparticles (termed LCNPs for lipid crystalline nanoparticles) were composed of BJO and DOX plus monoolein and oleic acid, and they exhibited pH-dependent morphological properties, including inverted hexagonal, cubic, and emulsified microemulsion phases at pH 7.4, 6.8, and 5.3, respectively (Figure 5C). The LCNPs had an average diameter of around 180 nm.

The pH range around which the LCNPs exhibited morphological changes was relevant to the conditions found near and in tumor microenvironments, and the rate of in vitro release of DOX from the dual-loaded DOX-BJO LCNPs depended on the solution pH. An appreciably quicker release rate was observed at pH 5.3 vs. pH 7.4, and it was noted that the presence of a liquid crystalline structure tended to slow down the drug release (Figure 5D). In vitro cell cytotoxicity testing against a human breast cancer cell line revealed that blank LCNPs had no inhibitory activity, while LCNPs containing DOX and BJO had a more potent inhibitory activity than free DOX and BJO in combination without the nanocarrier. Importantly, the LNCPs also enabled inhibition against previously DOX-resistant cancer cells.

### 3.4. Spongosomes

There has also been an ongoing exploration of sponge-type nanocarriers that are called spongosomes, which are liquid crystalline lipid nanocarriers that exhibit a high encapsulation efficiency and controlled release, and that can protect active ingredients from degradation. Spongosomes typically lack a solid inner core structure, which allows them to function as spongy materials, and they are endowed with stabilizing molecules on the outer surface to support colloidal stability [80]. Compared to liposomal carriers that also lack a solid, inner core and are filled with an aqueous medium, a useful advantage of the sponge-like architecture of spongosomes is that it can encapsulate a wider range of drugs with varying hydrophilic and hydrophobic properties.

Zou et al. reported the fabrication of BJO-loaded spongosomes and conducted a detailed structural characterization using cryogenic TEM (Cryo-TEM), SAXS, and dynamic light scattering (DLS) techniques [81]. The spongosomes had sizes in the range of 120 to 200 nm in diameter. Due to the alginate coating, the colloidal properties of each spongosome sample were stable over a 30-day storage period. From a drug delivery perspective, it was noted that spongosomes containing 2 mg/mL BJO maintained a high encapsulation efficiency of around 90% even after 30 days. In vitro cancer cell testing showed that the spongosomes containing 2 mg/mL BJO had the most potent inhibitory effect. The IC_50_ values of free BJO and spongosomes containing 2 mg/mL BJO were around 68 and 28 μg/mL, respectively, while the spongosome without BJO was nontoxic.

**Figure 6:**
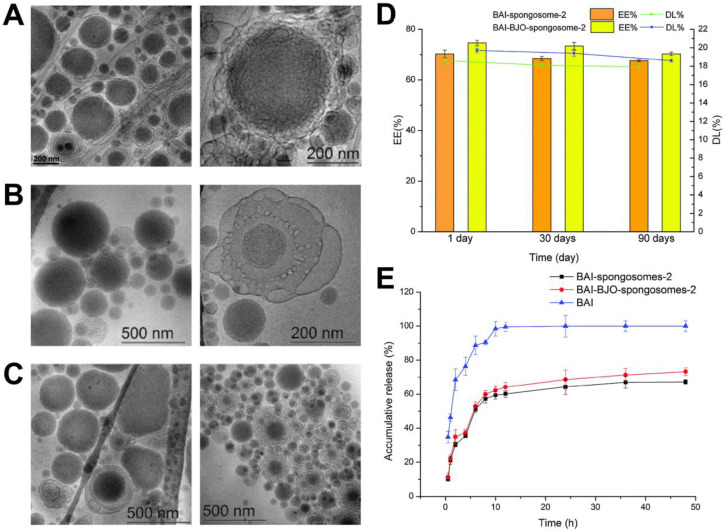
Cryo-TEM images are presented for spongosomes (**A**) without baicalin (BAI) or *Brucea javanica* oil (BJO), (**B**) with BAI only, and (**C**) with BAI and BJO; (**D**) Encapsulation efficiency (EE) and drug-loading (DL) values of the spongosomes containing BAI only or both BAI and BJO after different storage time periods postfabrication; (**E**) In vitro release profiles of BAI in phosphate-buffered saline (PBS) for spongosomes containing BAI only or both BAI and BJO, as compared to free BAI. Adapted with permission from [80].

In another effort, Chen et al. reported the development of poly(ethylene glycol)-coated spongosomes that encapsulated both BJO and baicalin (BAI), which is a small-molecule flavonoid with anticancer properties [80]. The coloaded spongosomes had a relatively large polydispersity, with the two main populations of sponges exhibiting around 60- and 310-nm diameters, respectively. Cryo-TEM images of spongosomes without drug, spongosomes containing BAI only, and spongosomes containing both BJO and BAI are presented in Figure 6A–C, and they support that the internal region of the spongosomes consisted of sponge-like lipid assemblies. In the presence of BJO, the encapsulation efficiency and drug-loading values of BAI were maintained at around 70% and 19%, respectively, and remained stable over time (Figure 6D). The spongosome vehicle also helped ensure the controlled release of BAI, while the inclusion of BJO had a modest tendency to improve the amount of released BAI (Figure 6E). Importantly, the dual-loaded spongosomes containing both BJO and BAI also demonstrated a superior inhibition of cancer cells in vitro as compared to spongosomes containing either drug alone.

## 4. Conclusions and Outlook

While there has long been interest in harnessing the medicinal properties of BJO and its molecular components in order to treat a wide range of medical diseases, recent progress in nanomedicine strategies has greatly advanced the field by realizing the potential to not only improve the solubility of BJO and its molecular components but also to achieve greater performance outcomes in terms of treatment efficacy and pharmacological properties. To date, a large number of proof-of-concept studies have been performed with many different types of nanoparticle-like delivery vehicles, and the results have set important precedents, especially with nanoemulsions. Table 1 summarizes the current status of technology development for different nanoparticle carrier types.

At the same time, there is an outstanding need to continue translational efforts by focusing on specific nanomedicine strategies that have the best potential for targeted applications and envisioned administration routes. For example, a suitable nanoparticle delivery vehicle for oral administration possibly has distinct properties from a suitable one for intravenous administration. Future efforts directed at creating nanomedicine delivery strategies that incorporate molecular components of BJO in a biomimetic manner would be useful, along with a deeper focus on characterizing and optimizing the physicochemical properties of specific nanoparticle-like delivery vehicles. Such work is needed in order to ultimately translate promising nanomedicines into viable clinical treatment options, and additional preclinical research directed at defining acute toxicity and other relevant pharmacological parameters through dose-escalation studies in animal models would also be beneficial as a key step towards eventual human clinical trials. Considering the clinical importance of BJO and emulsion-type BJOE systems already demonstrated in certain parts of the world, there is an enormous worldwide potential to continue pursuing nanomedicine delivery strategies to enhance the prospects of BJO and its molecular components as viable treatment options for a wide range of medical conditions, including various types of cancer and anti-inflammatory diseases.

There is also a significant opportunity to further explore how nanomedicine formulations of quassinoids might be used treat additional types of diseases, since quassinoids have been shown potentially useful in treating neurodegenerative diseases such as spinal muscular atrophy [82], in stimulating lipolysis in adipocyte cells that could be useful for combating obesity [83], in inhibiting insect pests [84], and in treating parasitic infections [85]. Since quassinoids are found in a wide range of plants, the nanomedicine delivery strategies discussed herein could be further explored to encapsulate various types of seed oils and purified quassinoids with distinct medicinal activities. Such possibilities would open the door to developing broadly effective nanomedicine delivery strategies that could combine the benefits of naturally evolved molecules with targeted biological functions and nanoparticle carriers with tunable structural properties in order to realize new application opportunities across human medicine, biotechnology, and agricultural science.

## Figures and Tables

**Table 1:** Status of different nanomedicine delivery strategies under development for BJO and/or purified quassinoid encapsulation.

Nanomedicine Class	Development Status	Main Effects
Nanoemulsions	In vivo treatment efficacy in mouse and rat models	- Loaded BJO or pure quassinoid in nanoemulsions with a 20- to 40- nm diameter, and could be developed into liquid or solid formulations. - BJO nanoemulsions had a reduced toxicity in vitro and side effects in vivo compared to BJOE, along with a greater bioavailability and circulation time. - BJO nanoemulsions had a greater treatment performance against cancer cells in vitro and mouse tumors in vivo than BJOE, as well as an improved anti-inflammatory performance in vivo.
Liposomes	In vivo treatment efficacy in mouse model	- BJO liposomes with a ~110-nm diameter had a lower toxicity and greater circulation time in mice than BJOE. - BJO liposomes had a greater treatment performance against cancer cells in vitro and mouse tumors in vivo than BJOE.
Nanostructured Lipid Carriers	In vitro drug release and cancer cell inhibition	- Loaded BJO with stable encapsulation in ~180-nm diameter nanostructures, pH-dependent changes in morphological structure, and slow drug release. - Loaded BJO had a greater inhibitory potency than free BJO and induced cancer cell apoptosis.
Spongosomes	In vitro drug release and cancer cell inhibition	- -Loaded BJO with stable encapsulation in 60- to 300-nm diameter nanostructures. - -BJO in spongosomes had a greater inhibitory activity than free BJO.
